# Classification methods of pulmonary contusion based on chest CT and the association with in-hospital outcomes: a systematic review of literature

**DOI:** 10.1007/s00068-024-02666-w

**Published:** 2024-09-10

**Authors:** Max R. Van Diepen, Mathieu M. E. Wijffels, Michael H. J. Verhofstad, Esther M. M. Van Lieshout

**Affiliations:** Trauma Research Unit Department of Surgery, Erasmus MC, University Medical Center Rotterdam, P.O. Box 2040, 3000 CA Rotterdam, The Netherlands

**Keywords:** Pulmonary contusion, Rib fractures, Chest trauma, Computed tomography

## Abstract

**Introduction:**

Patients sustaining pulmonary contusion (PC) have a higher risk of complications and long-term respiratory difficulty. Computed tomography (CT) scans have a high sensitivity for PC. However, since PC develops over time, CT scans made directly post-trauma may underestimate the full extent of PC. This creates a need to better define in which PC-patients complications are more likely. The aim of this systematic review was to identify different classification systems of PC, and investigate the association between amount of PC and in-hospital outcomes.

**Methods:**

A systematic review was conducted in accordance with PRISMA guidelines. Studies reporting a classification system for PC after blunt thoracic trauma based on a CT scan were included. Outcomes were classification method of PC and the relation between classification and pulmonary complications and in-hospital outcomes.

**Results:**

Twenty studies were included. Total number of patients ranged from 49 to 148,140 patients. The most common classification system used was calculating the percentage of contused lung volume. Other classification methods were based on Blunt Pulmonary Contusion score-6 and -18, Abbreviated Injury Score and Thoracic Trauma Severity scores. Worse outcomes were generally associated with between > 18 to > 24% contusion volume.

**Discussion:**

The heterogeneity of currently available literature makes comparing classification methods challenging. The most common classification of PC was based on volumetric analysis. Calculating a percentage of PC as part of the total volume allows for the highest level of segmentation of lung parenchyma as compared to using BPC-6, BPC-18, or AIS. Contusion volume exceeding 18–24% was generally associated with worse outcomes.

**Supplementary Information:**

The online version contains supplementary material available at 10.1007/s00068-024-02666-w.

## Introduction

Pulmonary contusion (PC) is a frequent and serious injury following blunt chest trauma. PC occurs in approximately 17% of multiply injured patients (Injury Severity Score > 15) [1] and between 25 and 30% of all patients who have sustained blunt chest trauma [2]. Patients with PC have higher risk of adverse events such as pneumonia and acute respiratory distress syndrome (ARDS), sometimes requiring mechanical ventilation. Additionally, patients suffering PC may develop long-term respiratory sequelae [3]. Respiratory problems as a result of PC usually resolve within 3–5 days, but delayed deterioration is possible [4].

PC is primarily diagnosed using radiologic modalities. Traditionally, the diagnosis is based on plain chest radiographs (CXR), but ultrasound and computed tomography (CT) scans have demonstrated superior detection [4, 5]. Since PC develops over time, it may be missed if only a single modality is used too early or too late. The severity of PC is underestimated on initial CXR, and the full extent of PC may not become visible on CXR until 48 h after jury, lagging behind clinical signs such as hypoxia and shortness of breath [2]. CT scans have a very high sensitivity for PC, however not all PC seen on CT lead to respiratory dysfunction [4, 6]. Also, radiographic worsening of contusion on a repeated CT scan, performed at median 85 h after trauma, was not associated with a worse clinical outcome [7]. As the threshold to perform a CT scan to evaluate trauma patients is very low nowadays it can be assumed that PC, both clinically significant and insignificant, is detected more often. This creates a need to define which changes suspect for PC on CT scans will lead to worse outcomes. The aim of this systematic review was to identify the different classification systems for PC used in literature based on chest CT in the acute setting in patients who sustained blunt thoracic trauma, and to determine the association between the extent of PC measured per system and in-hospital outcomes.

## Methods

### Search strategy

A protocol was written before initiation of the study. This systematic review was conducted using the Preferred Reporting Items for Systematic Reviews and Meta-Analyses (PRISMA) guidelines [8]. Databases Embase, Medline, Web of Science, and Cochrane Central were searched systematically for clinical studies describing outcomes of patients sustaining PC diagnosed on chest CT. The search strategies were adapted to accommodate the unique searching features of each database, including database-specific MESH and EMTREE controlled vocabulary terms. Searches were not limited by date, language, or publication status. The initial literature search was performed by a biomedical information specialist on November 11, 2021 and the search was renewed on March 8, 2024.

### Study selection

For inclusion, studies had to report a classification system for PC after blunt thoracic trauma based on a CT scan within 72 h after trauma, in a population consisting of at least 80% adults aged ≥ 18 years. Animal studies, meta-analyses, literature reviews, case reports, manuscripts not available in full text, and studies that did not report the relation between contusion and outcomes were excluded. No language criterion was used.

Titles and abstracts of the records were screened independently by two authors (MRVD and MMEW) for eligibility and any disagreement was resolved by consensus. The same procedure was used when reviewing the full text manuscripts of records deemed eligible based on title and abstract screening. A manual search of the reference lists of all included studies was performed in order to avoid missing any relevant publications.

### Quality assessment and evaluation of publication bias

The quality of all included studies was assessed using the methodological index for non-randomized trials (MINORS), which gives an ideal score of 16 for non-comparative studies and 24 for comparative studies [9]. The included records were evaluated independently by two authors (MRVD and MMEW) and disagreement was resolved by consensus. Publication bias was determined based on funnel plots.

### Outcome measures

Outcomes of interest were the methods used for classifying PC, how these classification systems measured the severity of contusion, and the relation between classification and severity, pulmonary complications (*i.e.* pneumonia and ARDS) and in-hospital outcomes (*i.e.* hospital- and intensive care unit length of stay (HLOS, ICU LOS), intubation, mechanical ventilation and duration of mechanical ventilation).

### Data collection

The following data were extracted from the included studies: biographic data of the publications such as author name and publication year, study period, study design, data on patient’s age and gender, injury severity score (ISS), number and laterality of PC, rib fractures, flail chest, pneumothorax, and hemothorax.

The method used to classify PC was extracted, as was the authors’ definition of severe PC. Parameters relevant to the extent of PC and associated with worse in-hospital outcomes and increased complications, such as HLOS, ICU-LOS, pneumonia, ARDS, intubation, mechanical ventilation, and duration of mechanical ventilation, were also extracted. Two authors (MRVD and MMEW) extracted the data, and any disagreement was resolved by consensus.

### Statistical analysis

A pooled analysis per subgroup – patients with versus without PC – was conducted for patient characteristics and injury characteristics using MedCalc Statistical Software version 18.2.1 (MedCalc Software bvba, Ostend, Belgium; http://www.medcalc.org; 2018). A generic inverse variance model was used for continuous data, and a proportions model was used for categorical data. Due to heterogeneity in study design across the studies, random effects models were used for data pooling. Pooled estimates (PE) with 95% confidence intervals (CI’s) were reported.

Based on the possible overlap of the 95% CI’s and pooled estimates, an evaluation can be made whether there is evidence of difference between the two pooled groups. There are three scenarios. First, if the 95% CI’s do not overlap, it is considered convincing evidence of a difference. Second, if the 95% CI’s overlap, but do not include both pooled estimates, there is inconclusive evidence of a difference. Lastly, if the 95% CI’s overlap and include both pooled estimates, there is no evidence of a difference [10].

## Results

### Search results

**Fig. 1:**
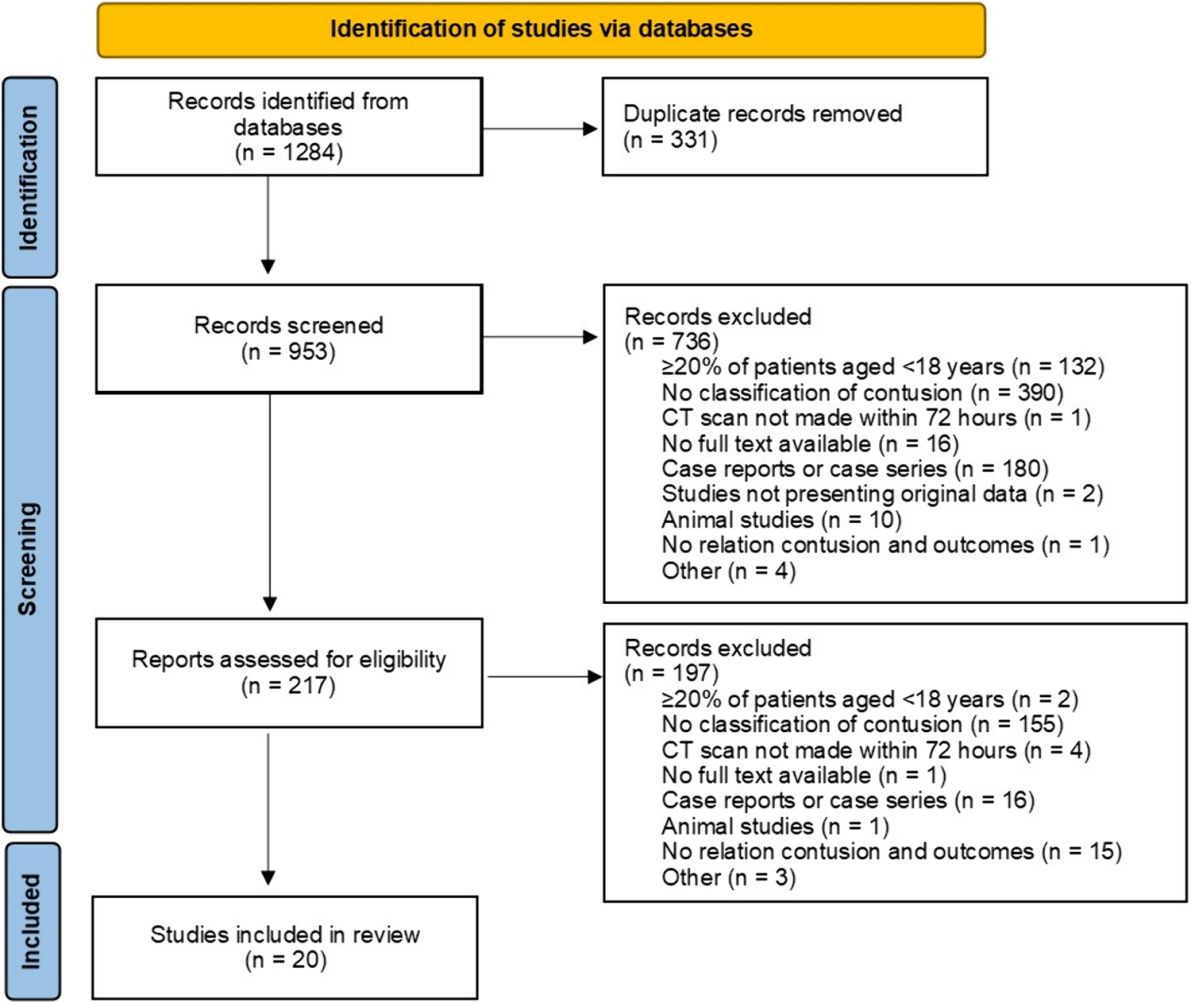
Study flowchart

A total of 1284 records were retrieved (828 from EMBASE, 204 from Medline, 242 from Web of Science, and 10 from Cochrane Central; Table 1). Figure 1 shows details on reasons for exclusion of identified records. After removal of duplicate records (n = 331), 953 records remained for eligibility screening. Of these 953 records, 736 were excluded based on title and abstract screening, leaving 217 records for full text analysis. In total, 20 studies remained for inclusion. A manual search of the references of the included studies yielded no new records. The most common reasons for exclusion were that studies did not report a classification system (n = 512), were case reports or case series (n = 187), or included ≥ 20% patients aged < 18 years (n = 129).

**Table 1:** Search strategy per database

Database	Total (n = 1284)	Deduplicated (n = 953)
Embase	828	666
Medline (ALL)	204	203
Web of Science	242	77
Cochrane Central	10	7

Embase: ('lung contusion'/exp OR (contusion/de AND 'lung injury'/de) OR ((lung OR pulmonary) NEAR/3 (contus*)):ab,ti) AND ('computer assisted tomography'/exp OR 'computed tomography scanner'/exp OR (((comput*) NEAR/3 (tomogra*)) OR MDCT):ab,ti OR ct:ti) AND ('predictive value'/exp OR prognosis/exp OR 'clinical outcome'/exp OR 'treatment outcome'/de OR mortality/exp OR fatality/de OR 'artificial ventilation'/exp OR (predict* OR grading OR progress* OR classif* OR quantitif* OR prognos* OR outcome* OR mortalit* OR ((artificial* OR mechanic*) NEAR/3 ventilat*) OR fatal*):ab,ti). Medline: ((Contusions/ AND Lung Injury/) OR ((lung OR pulmonary) ADJ3 (contus*)).ab,ti.) AND (exp Tomography, X-Ray Computed/ OR exp Tomography Scanners, X-Ray Computed/ OR (((comput*) ADJ3 (tomogra*)) OR MDCT).ab,ti. OR ct.ti.) AND (exp "Predictive Value of Tests" / OR exp Prognosis/ OR exp Treatment Outcome/ OR exp Mortality/ OR exp Respiration, Artificial/ OR (predict* OR grading OR progress* OR classif* OR quantitif* OR prognos* OR outcome* OR mortalit* OR ((artificial* OR mechanic*) ADJ3 ventilat*) OR fatal*).ab,ti.). Web of Science: TS = ((((lung OR pulmonary) NEAR/2 (contus*))) AND ((((comput*) NEAR/2 (tomogra*)) OR MDCT) OR ct) AND ((predict* OR grading OR progress* OR classif* OR quantitif* OR prognos* OR outcome* OR mortalit* OR ((artificial* OR mechanic*) NEAR/2 ventilat*) OR fatal*))). Cochrane Central: (((lung OR pulmonary) NEAR/3 (contus*)):ab,ti) AND ((((comput*) NEAR/3 (tomogra*)) OR MDCT):ab,ti OR ct:ti) AND ((predict* OR grading OR progress* OR classif* OR quantitif* OR prognos* OR outcome* OR mortalit* OR ((artificial* OR mechanic*) NEAR/3 ventilat*) OR fatal*):ab,ti)

### Quality assessment and evaluation of publication bias

The average MINORS score for the included comparative studies was 16 out of 24 (range 13–18) and 8 out of 16 (range 5–11) for non-comparative studies. Studies scored lowest on the items prospective data collection, unbiased assessment of the study endpoint, and prospective calculation of the study size, as well as baseline equivalent of groups for comparative studies. Table 2 provides a full overview of scores per MINORS item. As far as the sample sizes allow, the funnel plots did not raise concern for publication bias (supplementary data). Funnel plots of the No-PC group however had fewer studies, thus possibly not having sufficient power to detect possible publication bias.

**Table 2:** MINORS score per study

Study	Study design	Comparative or noncomparative	A clearly stated aim	Inclusion of consecutive patients	Prospective collection of data	Endpoint appropriate to the aim of the study	Unbiased assessment of the study endpoint	Follow-up period appropriate to the aim of the study	Loss of follow up less than 5%	Prospective calculation of the study size	Additional criteria for comparative studies				Total score*	
											An adequate control group	Contemporary group	Baseline equivalent of groups	Adequate statistical analysis		
															out of	
Becher et al*.* (2012) [1]	Retrospective	Noncomparative	2	2	0	2	0	1	2	0	N.A	N.A	N.A	N.A	9	16
Choi et al*.* (2021) [2]	Retrospective	Comparative	1	2	0	2	0	1	2	0	2	2	1	2	15	24
Choi et al*.* (2022) [3]	Retrospective	Noncomparative	2	2	0	2	0	2	1	0	N.A	N.A	N.A	N.A	9	16
Christin et al*.* (2003) [4]	Retrospective	Noncomparative	2	2	0	2	0	1	0	0	N.A	N.A	N.A	N.A	7	16
De Moya et al*.* (2011) [5]	Retrospective	Comparative	1	2	1	2	0	1	2	0	2	2	2	1	16	24
Deunk et al*.* (2010) [6]	Retrospective	Comparative	2	2	1	2	0	2	2	0	2	2	1	2	18	24
Hamrick et al*.* (2009)(7)	Retrospective	Noncomparative	2	2	0	2	0	1	0	0	N.A	N.A	N.A	N.A	7	16
Lee et al*.* (2023) [8]	Retrospective	Noncomparative	2	2	0	2	0	2	2	0	N.A	N.A	N.A	N.A	10	16
Li et al*.* (2021) [9]	Retrospective	Comparative	1	2	0	2	0	2	2	0	2	2	2	2	17	24
Mahmod et al*.* (2017) [10]	Retrospective	Noncomparative	2	1	0	2	0	2	2	0	N.A	N.A	N.A	N.A	9	16
Miller et al*.* (2001) [11]	Retrospective	Noncomparative	2	1	0	2	0	1	1	0	N.A	N.A	N.A	N.A	7	16
Miller et al*.* (2019) [12]	Retrospective	Comparative	2	2	0	2	0	2	2	0	2	2	1	2	17	24
Mommsen et al*.* (2012) [13]	Retrospective	Noncomparative	1	2	0	2	0	2	2	0	N.A	N.A	N.A	N.A	9	16
Negrin et al*.* (2017) [14]	Prospective	Noncomparative	2	1	1	2	0	2	1	0	N.A	N.A	N.A	N.A	9	16
Pal et al*.* (2017)(15)	Prospective	Noncomparative	2	2	1	2	0	2	2	0	N.A	N.A	N.A	N.A	11	16
Sarkar et al*.* (2023) [16]	Retrospective	Noncomparative	2	2	0	2	0	2	1	0	N.A	N.A	N.A	N.A	9	16
Sturmwassser et al*.* (2011) [17]	Retrospective	Noncomparative	2	1	0	2	0	1	0	0	N.A	N.A	N.A	N.A	6	16
Wang et al*.* (2011) [18]	Retrospective	Noncomparative	2	1	0	2	0	1	2	0	N.A	N.A	N.A	N.A	8	16
Wang et al*.* (2023) [19]	Retrospective	Noncomparative	0	2	0	2	0	1	0	0	N.A	N.A	N.A	N.A	5	16
Zingg et al*.* (2021) [20]	Retrospective	Comparative	1	2	0	1	0	1	1	0	2	2	1	2	13	24

N.A., not applicable

^*^Items are scored 0 (not reported), 1 (reported but inadequate) or 2 (reported and adequate)

### Study characteristics

Detailed study characteristics are shown in Table 3. Six studies compared patients with pulmonary contusion (PC) to patients without PC (no-PC) [11–16]. The remaining 14 studies only included patients with PC [17–30]. The exact timing at which initial and potential repeat CT scans were made were not reported in most studies. Most studies mentioned simply that admission CT’s were used. Two studies reported on CT’s made within 6 h, and four studies reported on CT’s made within 24–72 h after injury. All studies were developmental in nature, i.e. they developed novel models of PC classification.

**Table 3:** Overview of included studies classifying pulmonary contusion

Author (year)	Study period	Study design	Patients without pulmonary contusion			Patients with contusion			Laterality of contusion	
			Nr. of patients	Male sex	Age	Nr. of patients	Male sex	Age	Unilateral	Bilateral
Becher et al*.* (2012) [1]	N.D	Retrospective	N.A	N.A	N.A	202	141 (69.8%)	36 (17)	N.D	N.D
Choi et al*.* (2021) [2]	N.D	Retrospective	119,756	70,997 (59.3%)	64.5 (0.3)†	28,384	20,759 (73.1%)	50.3 (0.3)†	N.D	N.D
Choi et al*.* (2022) [3]	January 2010 – July 2020	Retrospective	N.A	N.A	N.A	332	257 (77.4%)	49 (31–62)‡	N.D	N.D
Christin et al*.* (2003) [4]	January 1998 – February 2000	Retrospective	N.A	N.A	N.A	49	38 (77.5%)	37.0 (17)	N.D	N.D
De Moya et al*.* (2011) [5] *Retrospective*	January 2002 –April 2007	Retrospective	149	98 (65.7%)	51.9 (21)	243	166 (68.3%)	45.0 (20.8)	120 (49.4%)	123 (50.6%)
De Moya et al*.* (2011) [5] *Prospective*	January 2002 –April 2007	Prospective	24	16 (66.7%)	45.6 (17.6)	31	19 (61.3%)	50.9 (20.3)	13 (41.9%)	18 (58.1%)
Deunk et al*.* (2010) [6] *CT only*	May 2005 – July 2008	Prospective	157	N.D	41 (16–92)	157	N.D	36 (16–85)	85 (54.1%)	72 (45.9%)
Deunk et al*.* (2010) [6] *CT* + *CXR*	May 2005 – July 2008	Prospective	N.A	N.A	N.A	98	N.D	36 (16–85)	58 (59.2%)	40 (40.8%)
Hamrick et al*.* (2009)(7)	January 2005 – May 2007	Retrospective	N.A	N.A	N.A	152	109 (71.7%)	33.6*	85 (55.9%)	67 (44.1%)
Lee et al*.* (2023) [8]	January 2019 – January 2020	Retrospective	N.A	N.A	N.A	73	56 (76.7%)	45.3 (15.9)	N.D	N.D
Li et al*.* (2021) [9]	January 2015 – December 2017	Retrospective	159	115 (72.3%)	39.0 (15.1)	151	116 (76.8%)	42.2 (15.9)	41 (27.2%)	110 (72.8%)
Mahmod et al*.* (2017) [10]	2012 – 2014	Retrospective	N.A	N.A	N.A	226	211 (93.4%)	35.2 (13.1)	87 (38.5%)	139 (61.5%)
Miller et al*.* (2001) [11]	July 1998 –January 2000	Retrospective	N.A	N.A	N.A	49	45 (91.8%)	N.D	14 (28.6%)	35 (71.4%)
Miller et al*.* (2019*)* [12] *Mild PC*	January 2011 – December 2014	Retrospective	1,126	761 (67.6%)	58.0*	236	116 (70.3%)	49.3*	N.D	N.D
Miller et al*.* (2019) [12] *Severe PC*	January 2011 – December 2014	Retrospective	N.A	N.A	N.A	85	69 (81.2%)	38.8*	N.D	N.D
Mommsen et al*.* (2012) [13]	January 2000 –December 2009	Retrospective	N.A	N.A	N.A	278	203 (73.0%)	42.7 (17.0)	N.D	N.D
Negrin et al*.* (2017) [14]	June 2011 –June 2015	Prospective	N.A	N.A	N.A	108	90 (83.3%)	41.3 (17.0)	45 (41.7%)	63 (58.3%)
Pal et al*.* (2017) [15]	October 2014 –October 2015	Prospective	N.A	N.A	N.A	62	43 (69.0%)	40.7 (14.2)	N.D	N.D
Sarkar et al*.* (2023) [16]	July 2016	Retrospective	N.A	N.A	N.A	280	222 (79%)	31 (22–49)‡	N.D	N.D
Sturmwassser et al*.* (2011) [17]	January 2005 –October 2010	Retrospective	N.A	N.A	N.A	106	74 (70.0%)	39.0 (1.8)	42 (39.6%)	64(60.4%)
Wang et al*.* (2011) [18]	January 2010 –October 2010	Retrospective	N.A	N.A	N.A	60	53 (88.3%)	40.13 (13.8)	14 (23.3%)	46 (76.7%)
Wang et al*.* (2023) [19]⁑	January 2014 – June 2020	Retrospective	N.A	N.A	N.A	515	381 (74.0%)	55.10	N.D	N.D
Zingg et al*.* (2021) [20] *AIS*	January 2014 –December 2018	Retrospective	211	144 (68.2%)	56.0 (45–66)‡	234	178 (76.1%)	41 (27–56)‡	N.D	N.D
Zingg et al*.* (2021) [20] *BPC-18*	January 2014 –December 2018	Retrospective	N.A	N.A	N.A	230	176 (76.5%)	41 (28–54)‡	N.D	N.D
Pooled estimate (95% CI)	N.A	N.A	N.A	66.2% (60.7.-71.5%)	50.3 (35.1–65.5)	N.A	75.4% (72.2–78.4%)	41.9 (38.1–45.6)	N.A	N.A

Data presented as n (%) or as mean (SD/range)

*AIS* abbreviated injury score, *BPC-18* blunt pulmonary contusion score 18, *95% CI* 95% confidence interval, *CT* computed tomography, *CXR* chest X-ray, *N.A.* not applicable, *N.D.* not described, *PC* pulmonary contusion

*No SD reported

⁑Values based on calculations of relative contribution per subgroup in the author’s analysis

^†^Standard error reported

^‡^Median (P_25_–P_75_) reported

Three of the comparative studies subdivided the PC group. Deunk et al*.* described one group where PC was only visible on CT and one group where PC was visible on both CXR and CT [13]. Miller et al*.* subdivided PC patients based on severity of PC, where mild PC was defined as 1–19% contused lung volume and severe PC was defined as ≥ 20% contused lung volume [15]. Zingg et al*.* included one group where PC was categorized based on the Abbreviated Injury Scale (AIS) and one group where PC was categorized based on the Blunt Pulmonary Contusion Score 18 (BPC-18) [16].

The study by De Moya et al*.* had both a retro- and prospective part. In the retrospective part, the authors identified predictors for the need for mechanical ventilation. The prospective cohort was subsequently used to test these predictors [12].

### Patient and injury characteristics

The total number of patients per study varied from 49 patients to 148,140 patients. The majority of patients with and without PC across all studies was male (66.2%, 95% CI 60.7–71.5% for no-PC patients and 75.4%, 95% CI 72.2–78.4% for PC patients), however the non-overlapping nature of the 95% CI’s is convincing evidence of difference between the two groups. The mean age ranged from 39.0 to 64.5 years for no-PC patients (50.3, 95% CI 35.1–65.5) and from 31.0 to 55.1 years for PC patients (41.9, 95% CI 38.1–45.6). Given that the 95% CI’s of the pooled estimates overlaps, but do not include both pooled estimates, there is inconclusive evidence of a difference in age between both groups. Between 40.8% and 76.7% of patients with PC had bilateral PC (Table 3).

Injury characteristics per study are detailed in Table 4. The mean ISS ranged from 8.3 to 29.9 for no-PC patients and from 13.3 to 34.0 for PC patients. Rib fractures and flail chest was reported in 37.5–100.0% and 0.0–3.8% for no-PC patients, and in 61.3–100.0% and 0.8–19.4% for PC patients. Pneumothorax was reported in 4.4–41.2% for no-PC patients and in 3.3–82.6% for PC patients. Hemothorax was reported in 0.0–28.9% for no-PC patients and in 3.4–90.3% for PC patients. Pooled estimates for each variable are shown in Table 4. In general, patients with PC had a higher ISS, more often a flail chest, pneumothorax, or hemothorax, and similar rates of rib fractures than patients without PC. The 95% CI’s of the pooled estimates for pneumothorax do not overlap, indicating a convincing evidence of a difference between both groups. However, for ISS, flail chest, and hemothorax, the 95% CI’s overlap but do not include the pooled estimate. This signifies that there is inconclusive evidence of a difference between the groups for these variables. For number of rib fractures, the 95%-CI’s overlapped and also included the pooled estimate, signifying no evidence of difference between groups. All pooled estimates were derived from Forrest plots, which are shown in the supplementary data.

**Table 4:** Injury characteristics

Author (year)	Patients without pulmonary contusion					Patients with pulmonary contusion				
	ISS	Rib fractures	Flail chest	Pneumo-thorax	Hemo-thorax	ISS	Rib fractures	Flail chest	Pneumo-thorax	Hemo-thorax
Becher et al*.* (2012) [1]	N.A	N.A	N.A	N.A	N.A	22 (18–29)‡	N.D	N.D	N.D	N.D
Choi et al*.* (2021) [2]	8.8 (0.1) †	119,756 (100.0%)	311 (0.3%)	20,754 (17.3%)	7,526 (6.3%)	14.0 (0.2)†	28,384 (100.0%)	237 (0.8%)	9,111 (32.1%)	2,518 (8.9%)
Choi et al*.* (2022) [3]	N.A	N.A	N.A	N.A	N.A	17 (13–26)‡	332 (100.0%)	17 (5.1%)	N.D	N.D
Christin et al*.* (2003) [4]	N.A	N.A	N.A	N.A	N.A	34 (17)	N.D	N.D	N.D	N.D
De Moya et al*.* (2011) [5] *Retrospective*	10.9 (5.9)	86 (57.7%)	1 (0.7%)	32 (21.5%)	15 (10.1%)	14.7 (6.2)	169 (69.5%)	8 (3.3%)	100 (41.2%)	50 (20.6%)
De Moya et al*.* (2011) [5] *Prospective*	8.3 (4.7)	9 (37.5%)	0 (0.0%)	3 (12.5%)	0 (0.0%)	13.3 (6.4)	19 (61.3%)	2 (6.5%)	9 (29.0%)	4 (12.9%)
Deunk et al*.* (2010) [6] *CT only*	21 (9–59)	N.D	N.D	35 (22.3%)	8 (5.1%)	26 (9–59)	N.D	N.D	68 (43.3%)	14 (8.9%)
Deunk et al*.* (2010) [6] *CT* + *CXR*	N.A	N.A	N.A	N.A	N.A	34 (14–75)	N.D	N.D	76 (77.6%)	27 (27.6%)
Hamrick et al*.* (2009)(7)	N.A	N.A	N.A	N.A	N.A	23.9*	97 (63.8%)	N.D	N.D	N.D
Lee et al*.* (2023) [8]	N.A	N.A	N.A	N.A	N.A	22.3 (8.9)	N.D	N.D	N.D	N.D
Li et al*.* (2021) [9]	29.9 (9.5)	159 (100.0%)	N.D	7 (4.4%)	7 (4.4%)	31.9 (10.0)	151 (100.0%)	N.D	5 (3.3%)	13 (8.6%)
Mahmod et al*.* (2017) [10]	N.A	N.A	N.A	N.A	N.A	20.5 (10.4)	N.D	N.D	N.D	N.D
Miller et al*.* (2001)^{Miller, 2001 #63}^	N.A	N.A	N.A	N.A	N.A	24.4*	35 (71.4%)	3 (6.1%)	N.D	N.D
Miller et al*.* (2019*)* [12] *Mild PC*	18.1 (17.6–18.6)§	1,126 (100.0%)	31 (2.8%)	N.D	54 (4.8%)	21.3 (20.3–22.4)§	236 (100.0%)	12 (5.1%)	N.D	8 (3.4%)
Miller et al*.* (2019) [12] *Severe PC*	N.A	N.A	N.A	N.A	N.A	28.2 (25.7–30.6)§	85 (100.0%)	14 (16.5%)	N.D	3 (3.5%)
Mommsen et al*.* (2012) [13]	N.A	N.A	N.A	N.A	N.A	28.7 (9.3)	N.D	N.D	N.D	N.D
Negrin et al*.* (2017) [14]	N.A	N.A	N.A	N.A	N.A	31.9 (11.1)	91 (84.2)	21 (19.4%)	51 (47.2%)	9 (8.3%)
Pal et al*.* (2017) [15]	N.A	N.A	N.A	N.A	N.A	N.D	58 (93.5%)	N.D	N.D	56 (90.3%)
Sarkar et al*.* (2023) [16]	N.A	N.A	N.A	N.A	N.A	17 (12–26)‡	N.D	N.D	N.D	N.D
Sturmwassser et al*.* (2011) [17]	N.A	N.A	N.A	N.A	N.A	28 (1.2)	N.D	N.D	N.D	N.D
Wang et al*.* (2011) [18]	N.A	N.A	N.A	N.A	N.A	26.0 (12.7)	45 (75.0%)	3 (5.0%)	N.D	N.D
Wang et al*.* (2023) [19]⁑	N.A	N.A	N.A	N.A	N.A	14.0	515 (100%)	N.D	N.D	N.D
Zingg et al*.* (2021) [20] *AIS*	27 (22–38)‡	N.D	8 (3.8%)	87 (41.2%)	61 (28.9%)	29.5 (22–41)‡	N.D	34 (14.5%)	192 (82.1%)	128 (54.7%)
Zingg et al*.* (2021) [20] *BPC-18*	N.A	N.A	N.A	N.A	N.A	33 (24–41%)‡	N.D	35 (15.2%)	190 (82.6%)	131 (57.0%)
Pooled estimate (95%-CI)	14.5 (7.0–21.928)	90.9% (97.6–98.0%)	1.6% (0.2–4.1%)	19.3% (11.6–28.5%)	8.0% (4.9–11.9%)	24.1 (18.9–29.2)	91.8% (83.4–97.4%)	8.2% (3.6–14.2%)	47.5% (30.6–64.9%)	22.4% (11.8–35.2%)

Data presented as n (%) or as mean (SD/range)

*AIS* abbreviated injury score, *BPC-18* blunt pulmonary contusion score 18, *95% CI* 95% confidence interval, *CT* computed tomography, *CXR* chest X-ray, *ISS* injury severity score, *N.A.* not applicable, *N.D.* not described, *PC* pulmonary contusion

*No SD reported

⁑Values based on calculations of relative contribution per subgroup in the author’s analysis

^†^Standard error reported

^‡^Median (P_25_–P_75_) reported

^§^Mean (95% CI) reported

### Outcome measures—classification systems and severity of contusion

Detailed outcomes on classification systems, in-hospital outcomes and complications can be found in Table 5. Fourteen studies calculated the percentage of contused lung volume based on a variety of different volumetric analysis methods [13–15, 17–23, 25, 27, 29, 30]. Sturmwasser et al*.* created a CT Volume Index (CTVI) based on pixel analysis of CT scans [28]. De Moya et al*.*, Zingg et al*.*, and Mommsen et al*.* all used, among others, BPC-18 [12, 16, 24]. Mommsen et al*.* also incorporated percentage lung volume, AIS, and the Thoracic Trauma Severity Score (TTS) [24]. Pal et al*.* calculated uninvolved lung volume [26], and Choi et al*.* looked at the presence or absence of unilateral and bilateral contusion [11].

**Table 5:** Details on classification and risk factors for in-hospital outcomes and complications

Author (year)	Classification of contusion	Severe contusion	HLOS	ICU-LOS	Pneumonia	ARDS	Intubation	Mechanical ventilation	Duration of mech. vent
Becher et al*.* (2012) [1]	% lung volume	N.D	≥ 24% contusion	N.D	≥ 24% contusion	≥ 24% contusion	N.D	N.D	≥ 24% contusion
Choi et al*.* (2022) [3]	% lung volume	N.D	Positive association	N.D	N.D	N.D	N.D	Positive association	N.D
Christin et al*.* (2003) [4]	% lung volume	N.D	N.D	N.D	N.D	N.D	N.D	N.D	N.D
Deunk et al*.* (2010) [6]	% lung volume	N.D	≥ 18% contusion	≥ 18% contusion	≥ 18% contusion	≥ 18% contusion	N.D	N.D	N.D
Hamrick et al*.* (2009) [7]	% lung volume	N.D	N.D	N.D	N.D	N.D	N.D	> 20% contusion	N.D
Lee et al*.* (2023) [8]	% lung volume	> 20% contusion	N.D	N.D	> 20% contusion	N.D	N.D	N.D	N.D
Li et al*.* (2021) [9]	% lung volume	N.D	Contusion present	Contusion present	N.D	Contusion present	N.D	Contusion present	Contusion present
Mahmod et al*.* (2017) [10]	% lung volume	N.D	N.D	N.D	N.D	≥ 20% contusion	N.D	N.D	≥ 20% contusion
Miller et al*.* (2001) [11]	% lung volume	≥ 20% contusion	N.D	N.D	≥ 20% contusion	≥ 20% contusion	N.D	N.D	N.D
Miller et al*.* (2019*)* [12]	% lung volume	≥ 20% contusion	≥ 20% contusion	Any contusion	1–19% contusion	N.D	N.D	N.D	≥ 20% contusion
Negrin et al*.* (2017) [14]	% lung volume	N.D	N.D	N.D	N.D	> 8.1% contusion	N.D	N.D	N.D
Sarkar et al*.* (2023) [16]	% lung volume	N.D	N.D	N.D	N.D	Positive association	N.D	N.D	N.D
Wang et al*.* (2011) [18]	% lung volume	N.D	N.D	N.D	N.D	≥ 21.5% contusion	N.D	N.D	N.D
Wang et al*.* (2023) [19]	% lung volume	N.D	N.D	N.D	≥ 22.0% contusion	N.D	N.D	N.D	N.D
Sturmwassser et al*.* (2011) [17]	CT Volume Index (CTVI)	N.D	> 20% CTVI	> 20% CTVI	> 20% CTVI	N.D	N.D	N.D	> 20% CTVI
De Moya et al*.* (2011) [5]	BPC6 and BPC18	N.D	N.D	N.D	N.D	N.D	N.D	BPC6 > 2 BPC18 > 5	N.D
Zingg et al*.* (2021) [20]	Chest AIS and BPC18	Chest AIS ≥ 3 or BPC18 ≥ 3	Chest AIS ≥ 3 or BPC18 ≥ 3	Chest AIS ≥ 3 or BPC18 ≥ 3	N.D	N.D	N.D	N.D	Chest AIS ≥ 3 or BPC18 ≥ 3
Choi et al*.* (2021) [2]	Presence or absence of unilateral/bilateral contusion	N.D	Any Contusion present	N.D	Bilateral contusion	N.D	Bilateral contusion	N.D	N.D
Mommsen et al*.* (2012) [13]	AIS, BPC18, %lung volume, thoracic trauma severity score	N.D	N.D	≥ 19% contusion, BPC-18 ≥ 2, TTS ≥ 2	N.D	≥ 19% contusion, BPC-18 ≥ 2, TTS > 9	N.D	N.D	≥ 19% contusion, BPC-18 ≥ 2, TTS ≥ 2
Pal et al*.* (2017) [15]	Uninvolved lung volume in liters	N.D	N.D	N.D	N.D	N.D	N.D	Uninvolved volume 1676L ± 360L	N.D

*AIS* abbreviated injury scale, *ARDS* acute respiratory distress syndrome, *BPC-18* blunt pulmonary contusion score 18, *CTVI* contusion volume index, *HLOS* hospital length of stay, *ICU-LOS* intensive care unit length of stay, *N.A.* not applicable, *N.D.* not described, *TTS* thoracic trauma severity score

Four studies reported a cut-off value for severe contusion, three of these were based on percentage lung contusion and one was based on BPC-18 and chest-AIS. Severe contusion was defined as either ≥ 20% contusion volume or a chest-AIS ≥ 3 or BPC-18 ≥ 3.

### Outcome measures—pulmonary complications and in-hospital outcomes

Patients with between ≥ 18% to ≥ 24% contused lung volume, chest-AIS ≥ 3 or BPC-18 ≥ 3 had increased HLOS compared to patients with less PC (Table 5) [13, 15–17, 28]. Similarly, ICU-LOS was longer in patients with ≥ 18–20% contused lung volume or with a chest-AIS ≥ 3, BPC-18 ≥ 2 or TTS ≥ 2 compared to patients with a lower contused lung volume [13, 16, 24, 28]. In most studies pneumonia was associated with between ≥ 18% to ≥ 24% contused lung volume [13, 17, 21, 28, 29]. One study found an association with pneumonia for patients with mild contusion, defined as 1–19% contused lung volume [15]. ARDS was associated with between > 8.1% to ≥ 24% contused lung volume, as well as BPC-18 ≥ 2 or TTS > 9 [13, 17, 22–25, 29]. Longer duration of mechanical ventilation was associated with between ≥ 19% to ≥ 24% contused lung volume, as well as BPC-18 ≥ 2 or TTS ≥ 2 [15–17, 22, 24, 28].

Four studies did not report specific thresholds related with outcomes [11, 14, 18, 27]. Li et al*.* found that presence of any contusion was associated with HLOS, ICU-LOS, ARDS, mechanical ventilation, and duration of mechanical ventilation [14]. Similarly, Choi et al*.* found that presence of any contusion was associated with HLOS, and that presence of bilateral contusion was associated with pneumonia and need for intubation [11, 31]. Sarkar et al*.* found a positive association between percentage contused volume and ARDS [27]. Similarly, Choi et al*.* found a positive association between contusion volume and increased HLOS and need for mechanical ventilation, where each higher quartile percentage contusion was associated with higher odds of these adverse outcomes [18].

## Discussion

In this systematic review, the results of studies classifying PC based on chest-CT scan were analyzed, focusing on the methods used to classify the extent of PC. Pooled estimates demonstrated inconclusive or no evidence of a difference in patient demographics between patients with and without contusion, with the exception of male sex and higher occurrence of pneumothorax in patients with PC, as may be expected. The most common described classification of PC was based on calculating the percentage of contused lung volume on CT scans [13–15, 17–23, 25, 27, 29, 30]. The included studies showed that between > 18% and > 24% contusion volume was generally associated with worse outcomes.

All classification systems divide the lung parenchyma into sections and award points for the amount of contusion per section. Percentage contusion volume calculations theoretically divide the lungs into 100 sections, one for each percentage point. The BPC-6 score divides the lungs into 6 sections. Each lung is divided into upper, middle and lower thirds, where each third can be awarded a point if contusion is present, for a maximum score of 6. The BPC-18 divides each lung into the same thirds, and each field can be awarded between 1 and 3 points. A score of 1 indicates mild contusion with up to 33% opacification of that field, a score of 2 indicates moderate contusion with 33–66% opacification, and a score of 3 indicates severe contusion with > 66% opacification [32]. The TTS is a composite scoring system, awarding 0–5 points to 5 components. One of these components is pulmonary contusion, where more points are awarded when more lobes or both lungs are affected [31]. The AIS is based on how many lung lobes are affected with major/minor contusion. In order to predict outcomes as accurately as possible, the classification system should also be as precise as possible. Theoretically, the higher the segmentation of the lung parenchyma, the more precise a classification can be. This means that a score based on percentage contusion (100 segments) may be more precise, and thus of more added value clinically, than for instance the BPC-18 score (18 segments).

As CT scans are increasingly performed on trauma patients, more PC are found than in the days that only CXR was used. Klein et al*.* demonstrated that PC seen only on CT and not on conventional radiographs, causes a change in clinical management in only 20–30% of cases [33]. Thus, PC not seen on CXR seems often not severe enough to affect outcomes. One of the disadvantages of only using CXR to diagnose PC is that CXR is not sensitive enough to detect early PC. CT-scans have a higher sensitivity for PC. Furthermore, an advantage of CT scans then is the fact that they can be used for volumetric analysis, and thus allow for identification of patients at increased risk of complications [33, 34].

This study should be interpreted with regard for some weaknesses. Firstly, all included studies were graded as low quality, with the maximum MINORS score for comparative studies being 18 out of 24 and 11 out of 16 for noncomparative studies. Secondly, although most studies calculated percentage of contused lung volume to determine the amount of lung parenchyma affected, varying methods were used for calculating the percentages and CT’s of varying time points were used. For instance, volume loss due to pneumothorax or atelectasis was not corrected for in every study. Similarly, most studies used admission CT’s whereas other studies stated they used CT’s made within 24–72 h. Thirdly, five studies used methods other than calculating percentage of contused lung volume. Specifically, AIS, TTS, BPC-18, BPC-16, and presence versus absence of contusion were used. Because few studies investigated these methods, it is difficult to compare the accuracy of these methods to methods calculating percentage contused lung parenchyma. ROC analysis could have been useful to compare the studies, however due to the heterogeneity of the included studies this would yield unreliable results and was thus not performed. Lastly, we cannot definitively rule out that the included studies used the same definitions for the outcomes we are interested in, which may result in slightly different interpretation of the data.

In order to compare accuracy of prediction clinical outcome, for AIS or BPC-18 versus percentage of contused lung, more research is required. These studies should focus on standardization of quantifying the percentage contused lung volume and comparing the predictive value of this to established methods such as the BPC-18 and AIS. Artificial Intelligence (AI) may play a major role in catalyzing the standardization process and has already shown promising results in previous studies [18, 27]. In addition, to get a more complete view of the pathophysiological process, a combination of biomarkers and radiologic visualization will probably achieve the best diagnostic value and ability to predict which patients are at higher risk of adverse outcomes. In addition, inter- and intra-observer studies may be informative to investigate agreement on extent of PC and predictiveness when using different classification methods. The heterogeneity of currently available literature is an issue also raised by previous studies [35]. The guideline of the German Society for Trauma Surgery recommends diagnostic and treatment strategies for PC, however the authors mention that there is no standard method used clinically to classify PC [35]. This might be due to an absence of comparative literature regarding the impact of different classification systems on treatment [35]. In line with results from this study, the guideline mentions a possible correlation between contusion volume measured on CT scans and clinical course of patients.

Another aspect to consider are the pathophysiological processes involved in pulmonary contusion. Biomarkers that can be measured in serum and bronchoalveolar lavage fluid might be helpful for a proper assessment of the severity of the PC. Where CT scans can only visualize the edema associated with contusion, which often lags behind processes on a cellular level, measuring levels of biomarkers can quantify the amount of damage on a microscopic level. Such markers have already been found for various stages of ARDS [36, 37].

In conclusion, there is much heterogeneity between methods used to classify PC. We recommend developing and implementing a standardized method of quantifying percentage of PC based on a volumetric analysis method in prospective and randomized studies.

## Supplementary Information

Below is the link to the electronic supplementary material.

Supplementary file1 (PDF 1708 KB)

## Data Availability

No datasets were generated or analysed during the current study.
