# Supplementary material for: Classification methods of pulmonary contusion based on chest CT and the association with in-hospital outcomes: a systematic review of literature
Source: Eur J Trauma Emerg Surg. 2024 Sep 10;50(6):2727–40. doi: 10.1007/s00068-024-02666-w (PMC11666754; doi:10.1007/s00068-024-02666-w)

## Supplementary data

# Classification methods of pulmonary contusion based on chest CT and the association with in-hospital outcomes: a systematic review of literature

Max R. Van Diepen, BSc[1], Mathieu M.E. Wijffels, MD PhD[1], Michael H.J. Verhofstad, MD PhD[1], Esther M.M. Van Lieshout, PhD MSc[1]

[1]Trauma Research Unit Department of Surgery, Erasmus MC, University Medical Center Rotterdam, Rotterdam, The Netherlands

**Corresponding author:**

Dr. E.M.M. van Lieshout, PhD MSc

Trauma Research Unit Department of Surgery

Erasmus MC, University Medical Center Rotterdam

P.O. Box 2040

3000 CA Rotterdam

The Netherlands

Mail: e.vanlieshout@erasmusmc.nl

Phone: +31.10.7031050

## A1. Forrest plot male sex No-PC

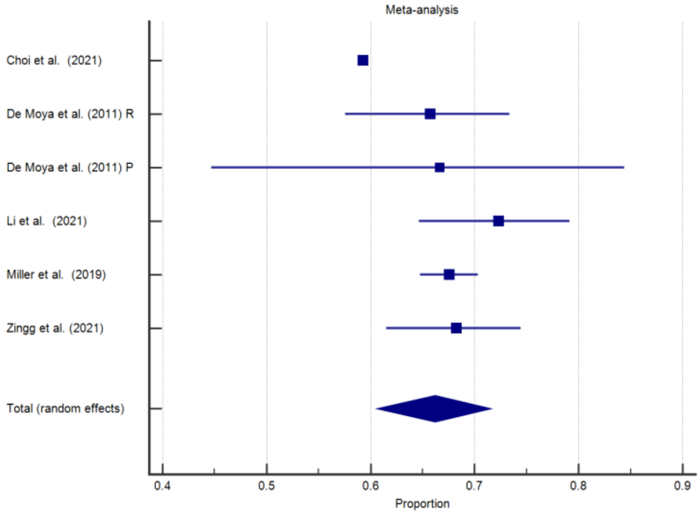


## A2. Forrest plot male sex PC

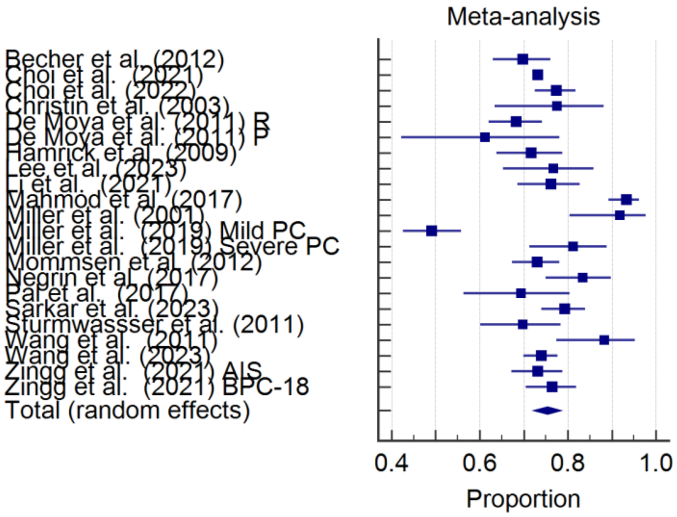


## B1. Forrest plot age No-PC

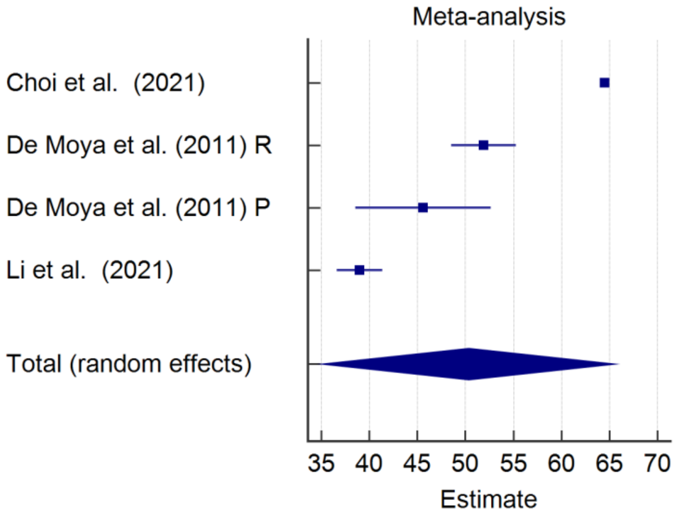


## B2. Forrest plot age PC

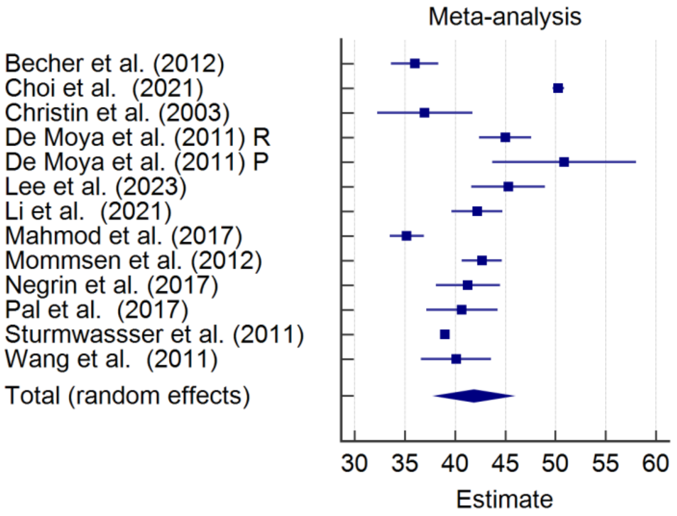


## C1. Forrest plot ISS No-PC

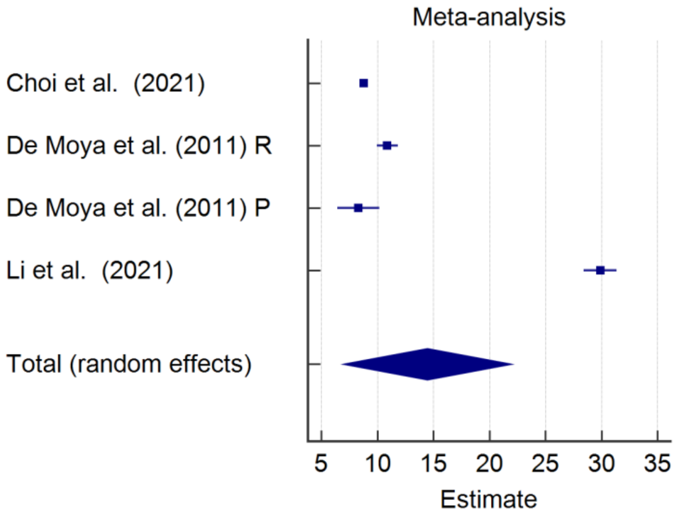


## C1. Forrest plot ISS PC

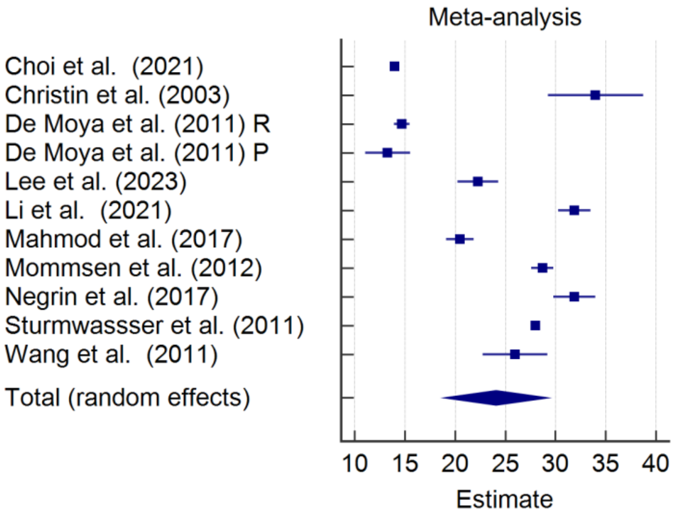

**D1. Forrest plot rib fractures No-PC**

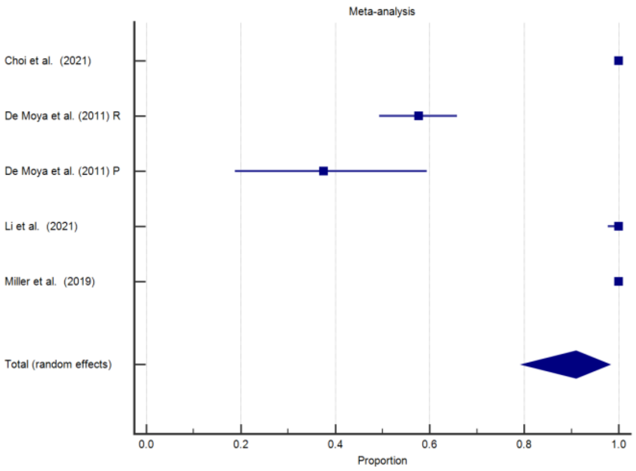


**D2. Forrest plot rib fractures PC**

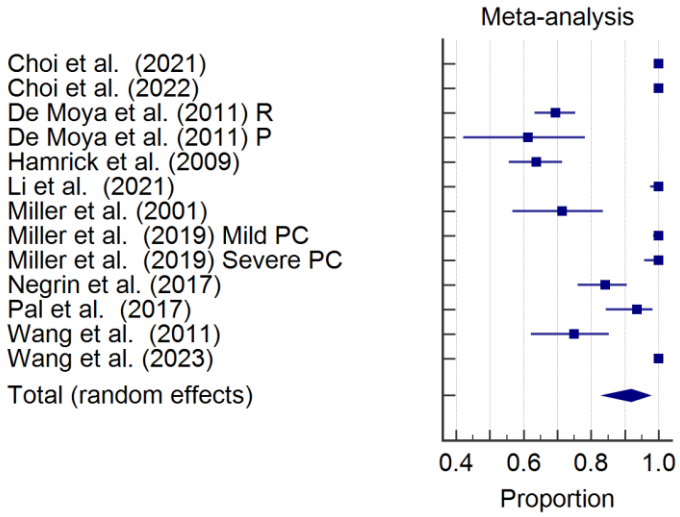


**E1. Forrest plot flail chest No-PC**

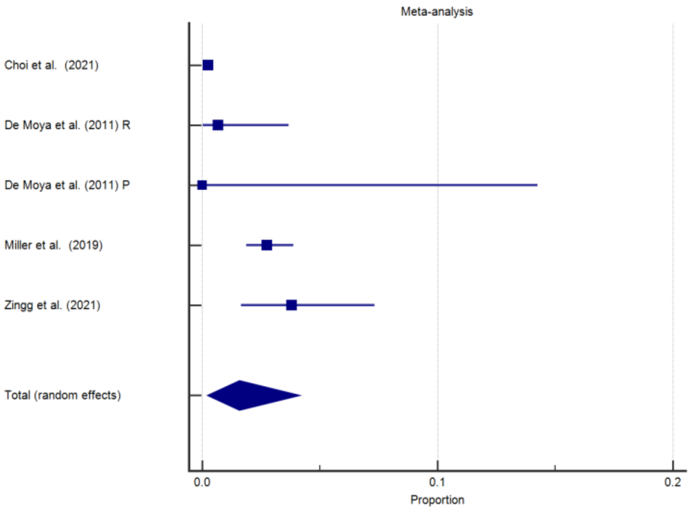


**E2. Forrest plot flail chest PC**

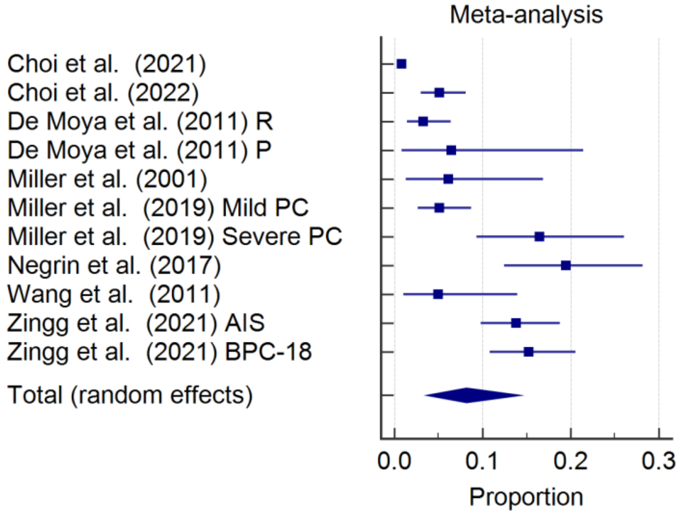


**F1. Forrest plot pneumothorax No-PC**

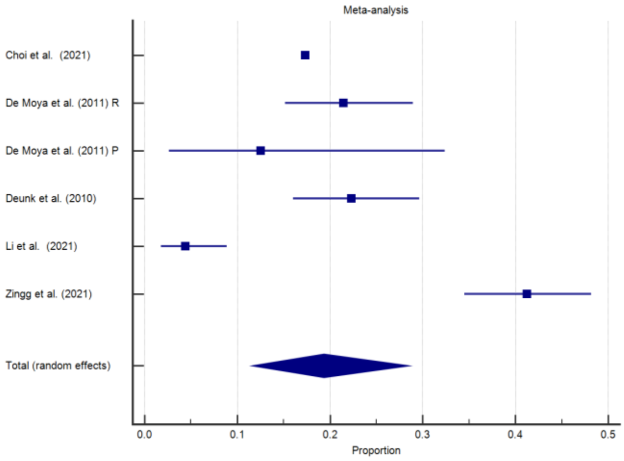


**F1. Forrest plot pneumothorax PC**

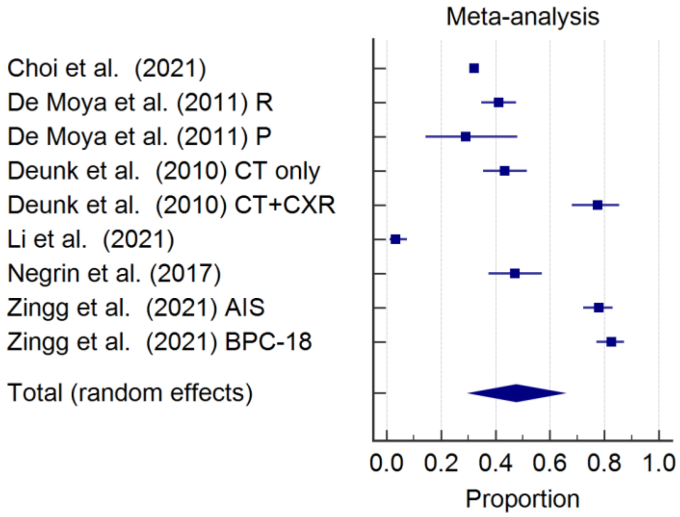

**G1. Forrest plot hemothorax No-PC**

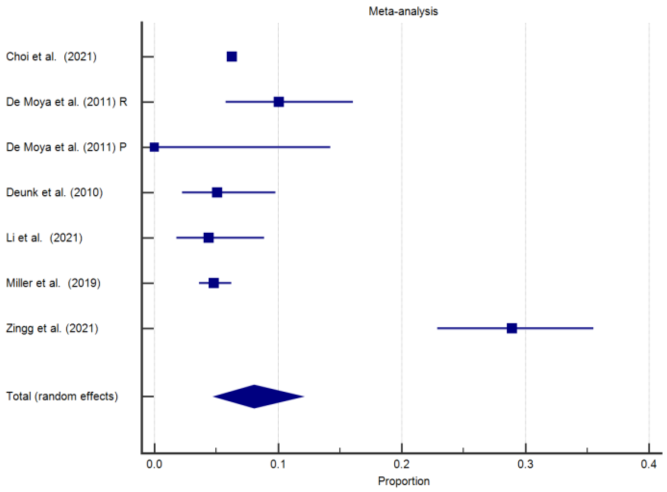


**G2. Forrest plot hemothorax PC**

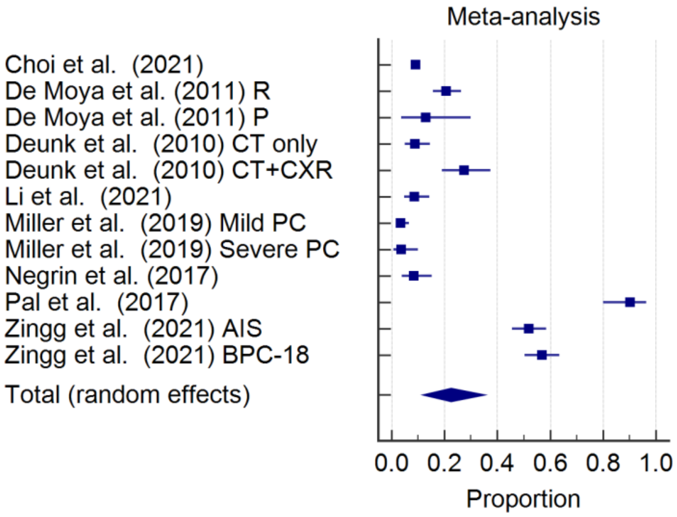

**A1. Funnel plot male sex No-PC**

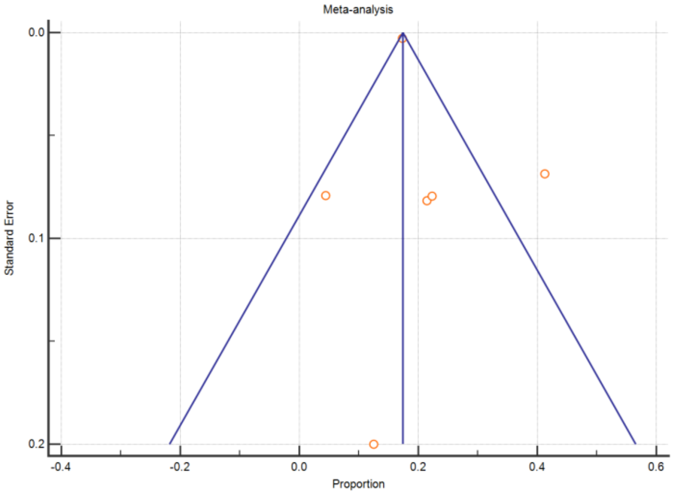


**A2. Funnel plot male sex PC**

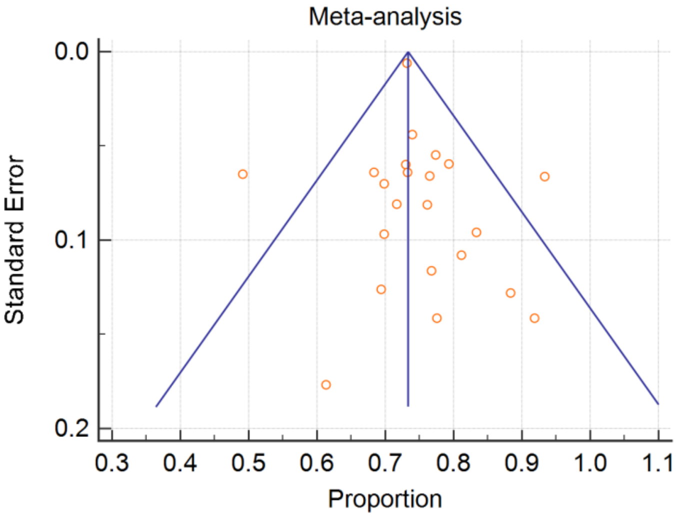


**B1. Funnel plot age No-PC**

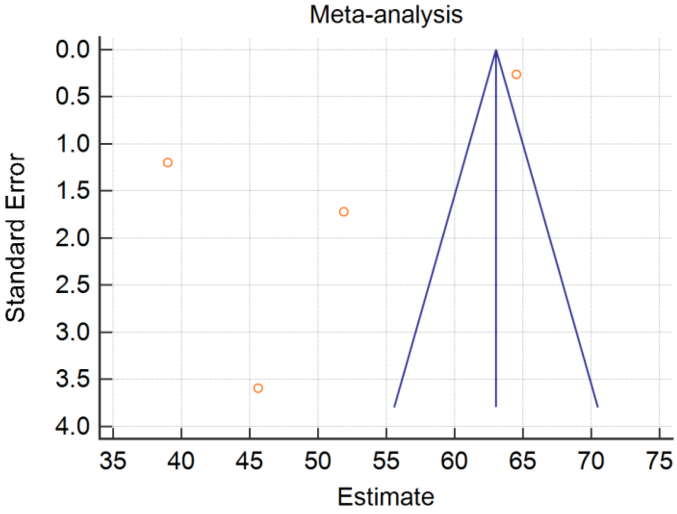


**B2. Funnel plot age PC**

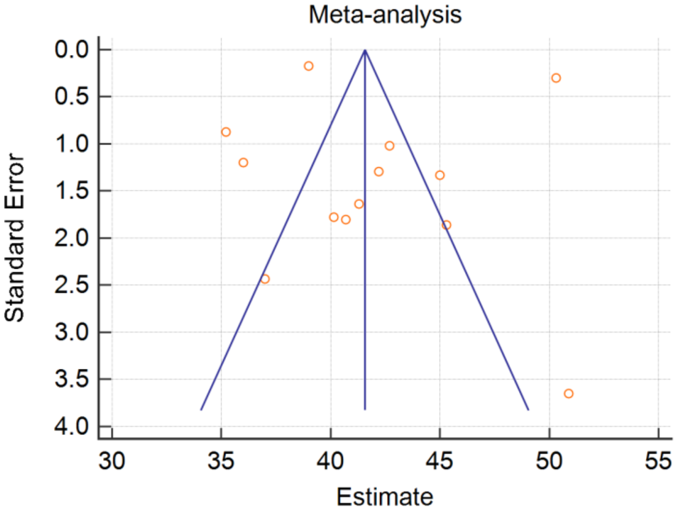


**C1. Funnel plot ISS No-PC**

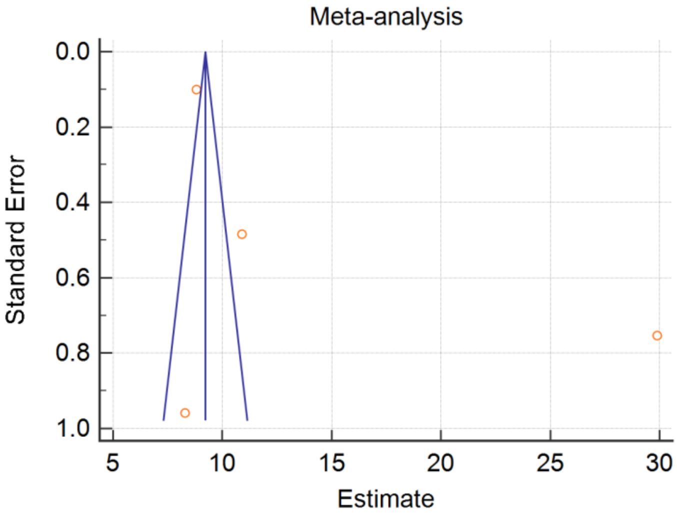


**C2. Funnel plot ISS PC**

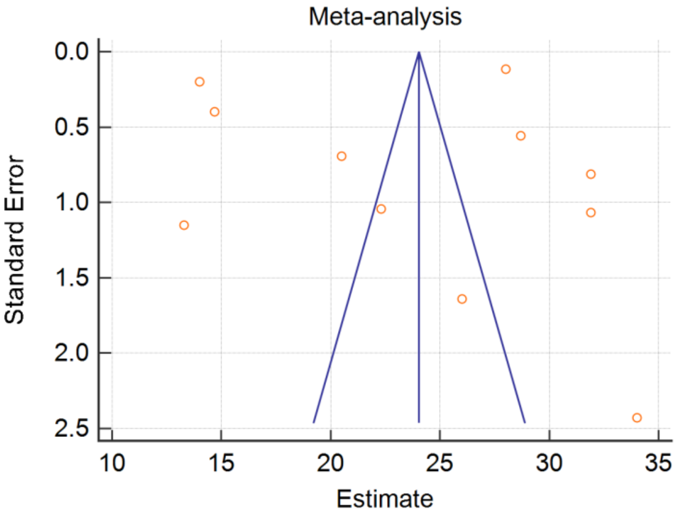

**D1. Funnel plot rib fractures No-PC**

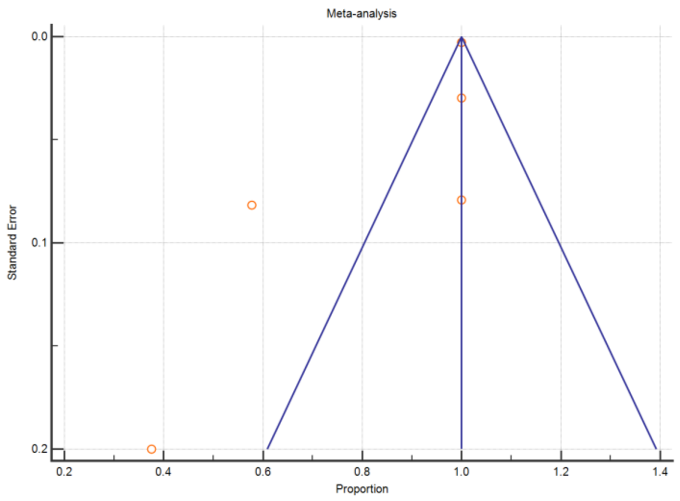


**D2. Funnel plot rib fractures PC**

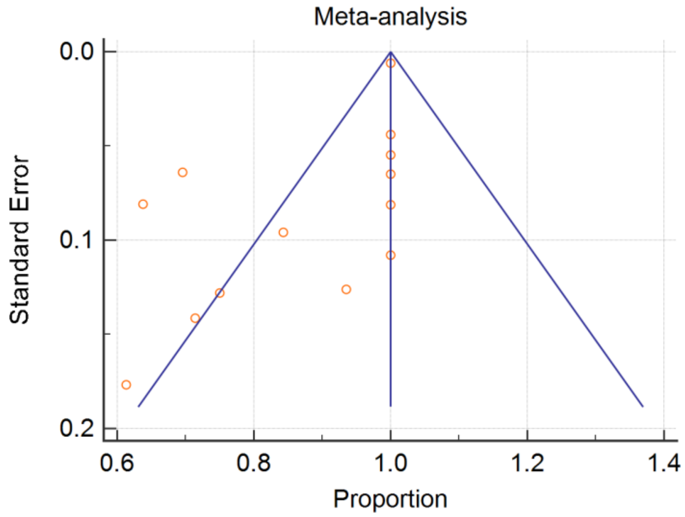


**E1. Funnel plot flail chest No-PC**

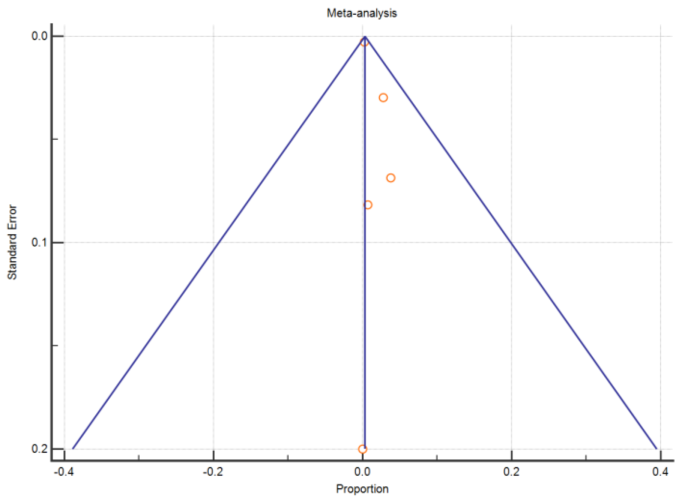


**E2. Funnel plot flail chest PC**

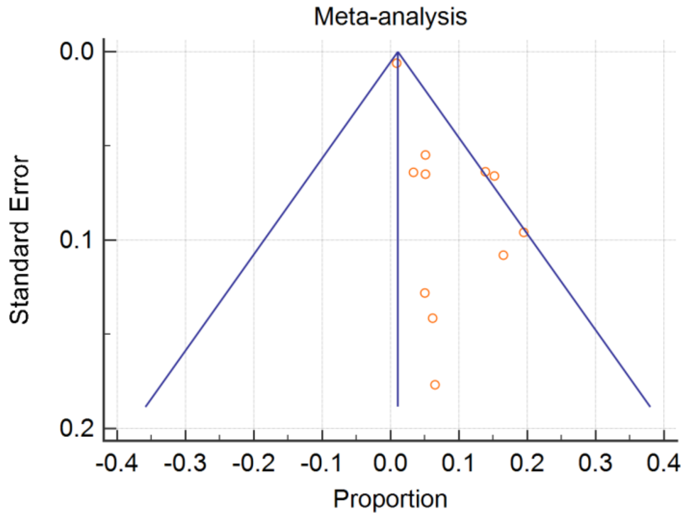


**F1. Funnel plot pneumothorax No-PC**

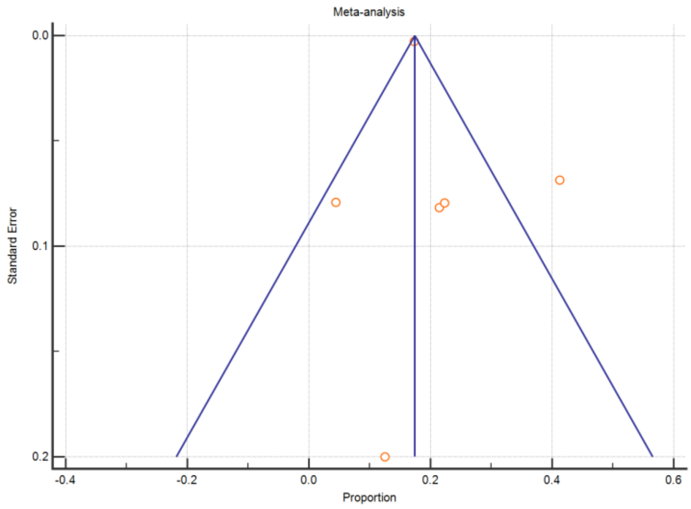


**F2. Funnel plot pneumothorax PC**

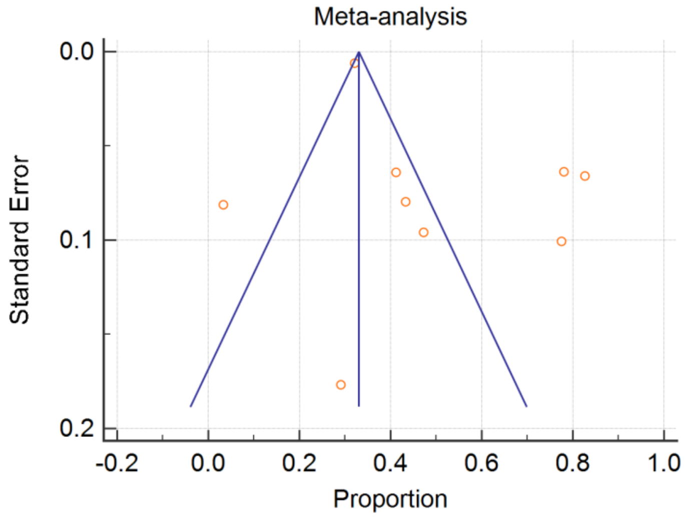

**G1. Funnel plot hemothorax No-PC**

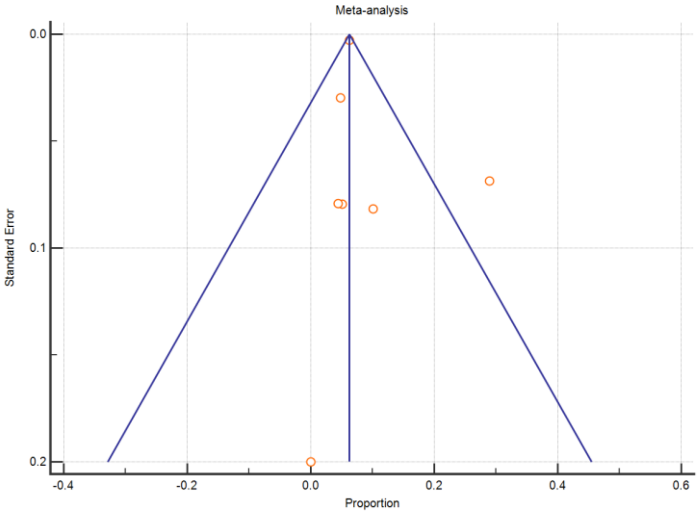


**G2. Funnel plot hemothorax PC**

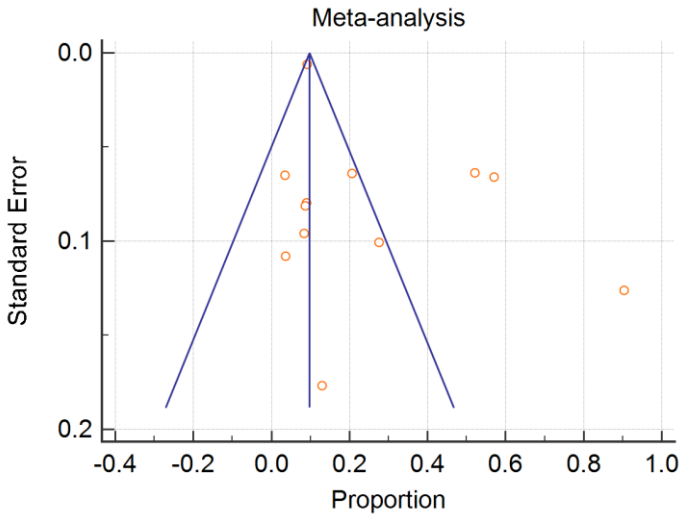

Supplement: Supplementary file 1 — Supplementary file1 (PDF 1708 KB) [file 68_2024_2666_MOESM1_ESM.pdf]
